# BIOSMILE: A semantic role labeling system for biomedical verbs using a maximum-entropy model with automatically generated template features

**DOI:** 10.1186/1471-2105-8-325

**Published:** 2007-09-01

**Authors:** Richard Tzong-Han Tsai, Wen-Chi Chou, Ying-Shan Su, Yu-Chun Lin, Cheng-Lung Sung, Hong-Jie Dai, Irene Tzu-Hsuan Yeh, Wei Ku, Ting-Yi Sung, Wen-Lian Hsu

**Affiliations:** 1 Institute of Information Science, Academia Sinica, Nankang, Taipei 115, Taiwan, PRoC; 2 Institute of Human Nutrition, Columbia University, New York, NY 10032, USA; 3 Biological Sciences & Psychology, Mellon College of Sciences, Carnegie Mellon University, Pittsburgh, PA, USA

## Abstract

**Background:**

Bioinformatics tools for automatic processing of biomedical literature are invaluable for both the design and interpretation of large-scale experiments. Many information extraction (IE) systems that incorporate natural language processing (NLP) techniques have thus been developed for use in the biomedical field. A key IE task in this field is the extraction of biomedical relations, such as protein-protein and gene-disease interactions. However, most biomedical relation extraction systems usually ignore adverbial and prepositional phrases and words identifying location, manner, timing, and condition, which are essential for describing biomedical relations. Semantic role labeling (SRL) is a natural language processing technique that identifies the semantic roles of these words or phrases in sentences and expresses them as predicate-argument structures. We construct a biomedical SRL system called BIOSMILE that uses a maximum entropy (ME) machine-learning model to extract biomedical relations. BIOSMILE is trained on BioProp, our semi-automatic, annotated biomedical proposition bank. Currently, we are focusing on 30 biomedical verbs that are frequently used or considered important for describing molecular events.

**Results:**

To evaluate the performance of BIOSMILE, we conducted two experiments to (1) compare the performance of SRL systems trained on newswire and biomedical corpora; and (2) examine the effects of using biomedical-specific features. The experimental results show that using BioProp improves the F-score of the SRL system by 21.45% over an SRL system that uses a newswire corpus. It is noteworthy that adding automatically generated template features improves the overall F-score by a further 0.52%. Specifically, ArgM-LOC, ArgM-MNR, and Arg2 achieve statistically significant performance improvements of 3.33%, 2.27%, and 1.44%, respectively.

**Conclusion:**

We demonstrate the necessity of using a biomedical proposition bank for training SRL systems in the biomedical domain. Besides the different characteristics of biomedical and newswire sentences, factors such as cross-domain framesets and verb usage variations also influence the performance of SRL systems. For argument classification, we find that NE (named entity) features indicating if the target node matches with NEs are not effective, since NEs may match with a node of the parsing tree that does not have semantic role labels in the training set. We therefore incorporate templates composed of specific words, NE types, and POS tags into the SRL system. As a result, the classification accuracy for adjunct arguments, which is especially important for biomedical SRL, is improved significantly.

## Background

The volume of biomedical literature available on the World Wide Web has experienced unprecedented growth in recent years. Processing literature automatically by using bioinformatics tools can be invaluable for both the design and interpretation of large-scale experiments. For this reason, many information extraction (IE) systems that incorporate natural language processing (NLP) techniques have been developed for use in the biomedical field. A key IE task in this field is the extraction of relations between named entities, such as protein-protein and gene-disease interactions.

Many biomedical relation-extraction systems use either cooccurrence statistics or sentence-level methods for relation extraction. Cooccurrence-based approaches extract biomedical relations by first tagging biomedical names and verbs in a text using dictionaries, and then identify cooccurrences of specific names and verbs in phrases, sentences, paragraphs, or abstracts. A variety of statistical tests, such as pointwise mutual information (PMI), the chi-square (*x*^2^), and the log-likelihood ratio (LLR) [1], have been used to decide whether a relation expressed by cooccurrences between a given pair really exists [2-6]. Sentence-level methods, on the other hand, usually consider only pairs of entities mentioned in the same sentence [7-9]. To detect and identify a relation, these systems generally use lexico-semantic clues inferred from the sentence context of the entity targets.

When extracting relations from complex natural language texts, both of the above approaches suffer from the same limitation in that they only consider the main relation targets and the verbs linking them. In other words, they frequently ignore phrases describing location, manner, timing, condition, and extent; however, in the biomedical field, these modifying phrases are especially important. Biological processes can be divided into temporal or spatial molecular events, for example activation of a specific protein in a specific cell or inhibition of a gene by a protein at a particular time. Having comprehensive information about when, where and how these events occur is essential for identifying the exact functions of proteins and the sequence of biochemical reactions. Detecting the extra modifying information in natural language texts requires semantic analysis tools.

**Figure 1:**
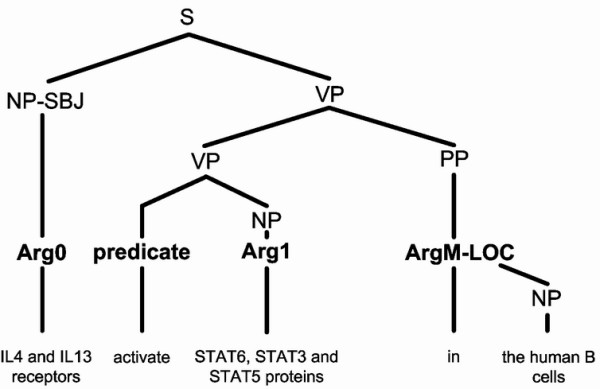
A parsing tree annotated with semantic roles.

*Semantic role labelling *(*SRL*), also called shallow semantic parsing [10], is a popular semantic analysis technique. In SRL, sentences are represented by one or more *predicate-argument structures *(*PAS*), also known as propositions [11]. Each PAS is composed of a predicate (e.g., a verb) and several arguments (e.g., noun phrases) that have different semantic roles, including main arguments such as an agent^1 ^and a patient^2^, as well as adjunct arguments, such as time, manner, and location. Here, the term *argument *refers to a syntactic constituent of the sentence related to the predicate; and the term *semantic role *refers to the semantic relationship between a predicate (e.g., a verb) and an argument (e.g., a noun phrase) of a sentence. For example, in Figure 1, the sentence "IL4 and IL13 receptors activate STAT6, STAT3, and STAT5 proteins in the human B cells" describes a molecular activation process. It can be represented by a PAS in which "activate" is the predicate, "IL4 and IL13 receptors" comprise the agent, "STAT6, STAT3, and STAT5 proteins" comprise the patient, and "in the human B cells" is the location. Thus, the agent, patient, and location are the arguments of the predicate.

Since SRL identifies the predicate and arguments of a PAS, it is also called predicate argument recognition [12]. In the natural language processing field, SRL has been implemented as a fully automatic process that can be operated by computer programs [13]. Given a sentence, the SRL task executes two steps: predicate identification and argument recognition. The first step can be achieved by using a part-of-speech (POS) tagger with some filtering rules. Then, the second step recognizes all arguments, including grouping words into arguments and classifying the arguments into semantic role categories. Some studies refer to these two sub-steps as *argument identification *and *argument classification*, respectively [14,15]. In the second step, it is often difficult to determine the word boundaries and semantic roles of an argument as they depend on many factors, such as the argument's position, the predicate's voice (active or passive) and the sense (usage).

SRL has been applied to information extraction because it can transform different types of surface texts that describe events into PAS'. In the newswire domain, Morarescu et al. [16] showed that, by incorporating semantic role information into an IE system, the F-score of the system can be improved by 15% (from 67% to 82%). This finding motivated us to investigate whether SRL could also facilitate information extraction in the biomedical field. In fact, most of the top open-domain SRL systems use machine-learning-based approaches [17-19]. However, at present, there is no large-scale machine-learning-based biomedical SRL system because of the lack of a sufficiently large annotated corpus for training.

In this paper, we propose an SRL system for the biomedical domain called BIOSMILE (BIOmedical SeMantIc roLe labEler). An annotated corpus and a PAS standard are essential for the construction of a biomedical SRL system. Considering our purpose is to train a machine learning SRL system, we use PropBank [20] and follow its annotation guidelines. Since PropBank must be annotated on a corpus containing full-parsing information (like a treebank, which is a collection of full parsing trees), we use the GENIA corpus, which includes 500 abstracts with full-parsing information. To evaluate SRL for use in the biomedical domain, we started with thirty verbs, which were selected because of their high frequency or important usage in describing molecular events. We employed a semi-automatic strategy using our previously created newswire SRL system SMILE (SeMantIc roLe labEler) [19] to tag a corpus derived from the GENIA corpus, and then asked human annotators with a background in molecular biology to verify the automatically tagged results. The resulting annotated corpus is called BioProp [21]. Lastly, we trained a biomedical version of SMILE on BioProp to construct an SRL system called BIOSMILE for the biomedical domain. To improve BIOSMILE's performance on adjunct arguments, which are phrases indicating the time, location, or manner of an event, we further exploit automatically generated patterns.

The corpus construction process is explained in the Background section, and the construction of our biomedical SRL system is described in the Methods section.

### Corpus selection

To construct BioProp, a biomedical proposition bank, we adopted GENIA [22] as the underlying corpus. It is a collection of 2,000 MEDLINE abstracts selected from the search results for queries using the keywords "human", "blood cells", or "transcription factors". GENIA is often used as a biomedical text mining test bed [23]. In its officially released version, it is annotated with various levels of linguistic information, such as parts-of-speech, named entities, and conjunctions. In the summer of 2005, Tateisi [24] published full parsing information for the corpus that basically follows the Penn Treebank II (PTB) annotation scheme [25] encoded in XML. The GENIA corpus annotated with full parsing information is called GENIA Treebank (GTB). Currently, GTB is a beta version containing 500 abstracts.

### Verb selection

As noted earlier, we chose thirty verbs because of their high frequency or important usage in describing molecular events. To select the verbs, we calculated the frequency of each verb based on its occurrence in GENIA, our underlying corpus, rather than in MEDLINE. It is noteworthy that some verbs that occur frequently in MEDLINE are rarely found in GENIA. Since we focus on molecular events, only sentences containing protein or gene names are used to calculate a verb's frequency. We listed verbs according to their frequency and removed generally used verbs such as is, have, show, use, do, and suggest. We then selected the verbs with highest frequencies and added some verbs of biological importance. The thirty verbs with their characteristics and frequency of occurrence in BioProp are listed in Table 1.

**Table 1:** The thirty selected verbs

Verb	Is the verb one of Top 30 frequent verbs in GENIA?	Is the usage different in the newswire and biomedical domains?	# of PAS's in BioProp
activate	Yes	Yes	145
affect	No	No	53
alter	No	No	27
associate	Yes	No	81
bind	Yes	Yes	189
block	No	No	56
decrease	No	No	41
differentiate	No	No	10
encode	Yes	Yes	75
enhance	Yes	No	37
express	Yes	Yes	186
increase	Yes	No	99
induce	Yes	No	263
inhibit	Yes	No	181
interact	No	Yes	34
mediate	Yes	No	103
modulate	No	Yes	22
mutate	No	Yes	5
phosphorylate	No	Yes	12
prevent	No	No	15
promote	No	Yes	13
reduce	No	No	38
regulate	Yes	No	116
repress	No	No	17
signal	No	No	7
stimulate	Yes	No	75
suppress	No	No	37
transactivate	No	Yes	21
transform	No	No	10
trigger	No	No	14

### PAS standard – Proposition Bank

To build our SRL system, we followed the PropBank I [20] standard. PropBank I, with more than ten years of development history, has a large verb lexicon, and contains more annotated examples than other standards [26]. In PropBank I, a PAS is annotated on top of a Penn-style full parsing tree. Figure 1 illustrates such a tree with syntactic and semantic role information. The semantic roles Arg0, Arg1, and ArgM-LOC are annotated on top of the words or phrases labelled as noun phrase subjects (NP-SBJ), noun phrases (NP), and prepositional phrases (PP), respectively. A proposition bank is a collection of full parsing trees annotated with propositions or PAS'. The first annotated PropBank-style proposition bank was the Wall Street Journal (WSJ) newswire corpus, which has 24 sections. Before being annotated with propositions, it was annotated with Penn-style full parsing trees. Sections 2 to 21 are conventionally used as a training set, Section 24 is used as a development set, and Section 23 is used as a test set in several NLP tasks [27].

PropBank I inherits verb senses from VerbNet, but the semantic arguments of individual verbs in PropBank I are numbered from 0. For a specific verb, Arg0 is usually the argument corresponding to the agent [28], while Arg1 usually corresponds to the patient or theme. For higher-numbered arguments, however, there is no consistent generalization for their roles. In addition to the main arguments, ArgMs refer to adjunct arguments. Table 2 details all the semantic role categories of arguments and their descriptions. The possible set of roles for a distinct sense of a verb is called a *roleset*, which can be paired with a set of syntactic frames that show all the acceptable syntactic expressions of those roles. A roleset with its associated frames is called a *frameset *[20]. Verbs may have different rolesets and framesets for different senses, which are numbered .01, .02, etc. An example of the frameset is given by the verb *activate *shown below.

**Table 2:** Argument types and their descriptions

**Type**	**Description**
Arg0	agent
Arg1	direct object/theme/patient
Arg2–5	not fixed
ArgM-NEG	negation marker
ArgM-LOC	location
ArgM-TMP	time
ArgM-MNR	manner
ArgM-EXT	extent
ArgM-ADV	general-purpose
ArgM-PNC	purpose
ArgM-CAU	cause
ArgM-DIR	direction
ArgM-DIS	discourse connectives
ArgM-MOD	modal verb
ArgM-REC	reflexives and reciprocals
ArgM-PRD	marks of secondary predication

Frameset **activate.01 **"make active"

Arg0: Activator

Arg1: Thing activated

Arg2: Activated-from

Arg3: Attribute

Ex1: [_Arg0 _IL4 and IL13 receptors] *activate *[_Arg1 _STAT6, STAT3, and STAT5 proteins] [_ArgM-LOC _in the human B cells].

Ex2: [_Arg1 _The simian virus 40 early promoter] is [_ArgM-DIS _also] [_ArgM-MNR _synergistically] *activated *[_Arg0 _by the Z/c-myb combination].

### Framesets of biomedical verbs

Basically, the annotation of BioProp is based on PropBank's framesets, which were originally designed for newswire texts. We further customize the framesets of biomedical verbs, since some of them may have different usages in biomedical texts. Table 1 indicates whether each verb has the same usage in the newswire and biomedical domains.

For verbs with the same usage in both domains, we adopt the newswire definitions and framesets. However, we need to make adjustments for other cases because some verbs have different usages and rarely appear in newswire texts. Thus, they are not defined in PropBank I. For example, "phosphorylate" is not defined in PropBank I, but it has been found increasingly in PubMed abstracts describing the experiment results of phosphorylated events [29]. Therefore, after analyzing every sentence in our corpus containing such verbs, we added the latter to our list and defined framesets for them. For verbs not found in PropBank I, but with similar usages to other verbs in the proposition bank, we borrowed the PropBank I definitions and framesets. For instance, "transactivate" is not found in PropBank I, but we can apply the frameset of "activate" to it.

Some verbs have unique biomedical meanings not defined in PropBank I; however, their usage is similar to verbs in Propbank I. In most cases, we borrow framesets from synonyms. For example, "modulate" is defined as "change, modify, esp. of music" in the PropBank I frame files. However, its usage is very similar to "regulate" in the biomedical domain. Thus, we can use the frameset of "regulate" for "modulate". Table 3 shows the framesets and corresponding examples of "modulate" in the newswire and biomedical domains, as well as those of "regulate" in PropBank I.

**Table 3:** Framesets and examples of "modulate" and "regulate"

**Predicate**	**Frameset**	**Example**
modulate (VerbNet)	**Arg0: **composer **Arg1: **music **Arg2: **from **Arg3: **to	[_Arg1 _The chords]*modulate*, but there is little filigree, even though his fingers begin to wander over more of the keys.
regulate (VerbNet)	**Arg0: **regulator **Arg1: **thing regulated	The battle focuses on [_Arg0_the state's certificate-of-need law], [_R-Arg0_which] *regulates *[_Arg1_investment in new medical technology].
modulate (BioProp)	**Arg0: **regulator **Arg1: **thing regulated	[_Arg0_Cytomegalovirus] *modulates *[_Arg1_interleukin-6 gene expression].

Some other verbs have different primary senses in the newswire and biomedical domains. "Bind", for example, is common in the newswire domain and usually means "to tie" or "restrain with bonds". In the biomedical domain, however, its intransitive use, "attach or stick to", is far more common. A Google search for the phrase "glue binds to" only returns 21 results, while the same search replacing "glue" with "protein" yields 197,000 hits. For such verbs, we just select the appropriate alternative meanings and corresponding framesets.

### Annotation of BioProp

Once the framesets for the verbs have been defined, we use a semi-automatic strategy to annotate BioProp. We used our newswire SRL system SMILE, which achieved an F-score of over 86% on Section 24 of PropBank I, to annotate the GENIA treebank automatically. Then, we asked three biologists to verify the automatically tagged results. One of the biologists has three years experience in biomedical text mining research, and he managed the task. The other two biologists received three months of linguistic training for this task. After annotating BioProp, we evaluated the performance of SMILE on BioProp. The F-score was approximately 65%, which is 20% lower than its performance on PropBank I. Even so, this semi-automatic approach substantially reduces the annotation effort.

### Inter-annotator agreement

We performed a preliminary consistency test on 1,982 instances of biomedical propositions by having two of the biologists annotate the results, while the third checked the annotations for consistency. Following the procedure used to calculate the inner-annotator agreement of PropBank [20], we measured the agreement between the two annotations before the adjudication step using the kappa statistic [30]. Kappa is defined with respect to the probability of inter-annotator agreement, *P*(*A*), and the agreement expected by chance, *P*(*E*), as follows:

κ=P(A)−P(E)1−P(E)
 MathType@MTEF@5@5@+=feaafiart1ev1aaatCvAUfKttLearuWrP9MDH5MBPbIqV92AaeXatLxBI9gBaebbnrfifHhDYfgasaacH8akY=wiFfYdH8Gipec8Eeeu0xXdbba9frFj0=OqFfea0dXdd9vqai=hGuQ8kuc9pgc9s8qqaq=dirpe0xb9q8qiLsFr0=vr0=vr0dc8meaabaqaciaacaGaaeqabaqabeGadaaakeaaiiGacqWF6oWAcqGH9aqpdaWcaaqaaiabdcfaqjabcIcaOiabdgeabjabcMcaPiabgkHiTiabdcfaqjabcIcaOiabdweafjabcMcaPaqaaiabigdaXiabgkHiTiabdcfaqjabcIcaOiabdweafjabcMcaPaaaaaa@3E07@

The inter-annotator agreement probability *P*(*A*) is the number of nodes that the annotators agree on the annotation divided by the total number of nodes considered. To calculate *P*(*E*), for each category *c*, let *p*_*1c *_denote the probability of *c *annotated by annotator 1, and *p*_*2c *_denote the probability annotated by annotator 2. Then P(*E*) is the summation of *p*_1c _* *p*_*2c *_over all categories c of the semantic role labels. However, the calculation of *P*(*A*) and *P*(*E*) is distinguished into two cases that correspond to role identification (role vs. non-role) and role classification, since the vast majority of arguments are located on a small number of nodes near the verb and we need to avoid inflating the kappa score artificially. For role identification, the denominator of *P*(*A*) and *P*(*E*) the total number of nodes considered, is given by the number of nodes in each parse tree multiplied by the number of predicates annotated in the sentence, and the numerator is given by the number of nodes that are labeled as arguments (without considering whether a correct argument is assigned). For the role classification kappa, we only consider nodes marked as arguments by both annotators, which yields the denominator of *P*(*A*) and *P*(*E*), and compute kappa over the choices of possible argument labels. Furthermore, for both role identification and role classification, we compute kappa to process ArgM labels in two ways. The first (denoted as "Including ArgM in Table 4) processes ArgM labels as arguments like any other type of argument, such that ArgM-TMP, ArgM-LOC and so on are considered as separate labels for the role classification kappa. In the second scenario (denoted as "Excluding ArgM in Table 4), we ignore ArgM labels, treating them as unlabeled nodes, and calculate the agreement for identification and classification of numbered arguments only.

**Table 4:** Inter-annotator agreement

		P(A)	P(E)	Kappa score
Including ArgM	role identification	.97	.52	.94
	role classification	.96	.18	.95
	combined decision	.96	.18	.95
Excluding ArgM	role identification	.97	.26	.94
	role classification	.99	.28	.98
	combined decision	.99	.28	.98

The kappa statistics for the above decisions are shown in Table 4. Given the large number of obviously irrelevant nodes, agreement on role identification is very high (.97 for both treatments of ArgM). The kappas for the more difficult role classification task are also high, .95 for all types of ArgM and .98 for numbered arguments only.

### Related work

Wattarujeekrit et al. [26] developed PASBio, which has become a standard for annotating predicate-argument structures in the biomedical domain. It contains analyzed PAS's for over 30 verbs and is publicly available. Using predicate argument structures to analyze molecular biology information, PASBio is specifically designed for annotating molecular events and defines a core argument as one that is important for completing the meaning of an event. If a locative argument appears in a specific molecular PAS with a frequency greater than 80%, it is considered necessary and is therefore a main argument. To describe molecular events in greater detail, PASBio places biomedical constraints on main arguments. For example, considering the verb "express", its Arg1, which is defined as named entity being expressed, is limited to a gene or gene products.

Shah et al. [31] successfully applied PASBio in the construction of the LSAT system for extracting information about alternative transcripts from the same gene, while Cohen et al. [32] showed that the suitability of the PAS representational model of representation for biomedical text. They concluded that PAS representations work well for biomedical text. Kogan et al. [33] built a domain-specific set of PAS for the medical domain. Their work agrees a bit more with ours in terms of their assessment of the match between PropBank's representations and the biomedical domain.

Unlike PASBio, BioProp is not a standard for annotating the PAS' of biomedical verbs. The main goal of BioProp is to port the proposition bank to the biomedical domain for training a biomedical SRL system. Thus, BioProp follows PropBank guidelines and uses the latter's framesets with further customization for some biomedical verbs. Subsequently, we use PropBank I as our initial training corpus for the construction of BioProp, and then ask annotators to refine the automatically tagged results. This semi-automatic approach substantially reduces the annotation effort so that Bioprop can be used for training SRL systems in the biomedical domain.

## Results and discussion

### Datasets

We use PropBank I and BioProp, which are associated with the general English and biomedical domains, respectively, as the sources of our data. The PropBank I corpus contains 950,028 words, 39,892 sentences, and 18,737 PAS'. However, only 1,449 of the PAS use the 30 biomedical verbs on our list as their predicates. BioProp currently contains 1,982 PAS'. The numbers and ratios of each argument type in the PAS' of the selected 30 biomedical verbs in PropBank I and BioProp are listed in Tables 5 and 6, respectively.

**Table 5:** Distribution of argument types in PropBank I

**Argument Type**	**Number**	**Ratio**
Arg0	897	23.96%
Arg1	1440	38.46%
Arg2	361	9.64%
Arg3	133	3.55%
ArgM-NEG	55	1.47%
ArgM-LOC	58	1.55%
ArgM-TMP	207	5.53%
ArgM-MNR	122	3.26%
ArgM-EXT	7	0.19%
ArgM-ADV	122	3.26%
ArgM-PNC	21	0.56%
ArgM-CAU	29	0.77%
ArgM-DIR	1	0.03%
ArgM-DIS	86	2.30%
ArgM-MOD	204	5.45%
ArgM-REC	1	0.03%

Total	3744	100.00%

**Table 6:** Distribution of argument types in BioProp

**Argument Type**	**Number**	**Ratio**
Arg0	1355	25.03%
Arg1	1961	36.22%
Arg2	313	5.78%
Arg3	10	0.18%
ArgM-NEG	103	1.90%
ArgM-LOC	377	6.96%
ArgM-TMP	141	2.60%
ArgM-MNR	477	8.81%
ArgM-EXT	23	0.42%
ArgM-ADV	301	5.56%
ArgM-PNC	3	0.06%
ArgM-CAU	15	0.28%
ArgM-DIR	22	0.41%
ArgM-DIS	179	3.31%
ArgM-MOD	121	2.23%
ArgM-REC	6	0.11%
ArgM-PRD	7	0.13%

Total	5414	100.00%

### SMILE and BIOSMILE

We use two SRL systems: SMILE [19] and BIOSMILE [34]. The main difference between them is that SMILE is trained on PropBank I, while BIOSMILE is trained on BioProp. In addition, BIOSMILE has additional biomedical-specific features. Details of the features and the statistical models used in the two systems will be introduced in the Methods section.

### Experiment design

We design two experiments: one to compare the performance of SMILE and BIOSMILE on biomedical applications by testing them on BioProp, and the other to measure the effects of using biomedical-specific features on the system's performance.

#### Experiment 1: improvement by using biomedical proposition bank

Since SMILE and BIOSMILE are trained on the corpora of different domains, in this experiment, we examine the improvement in the performance of the SRL system trained on a biomedical proposition bank. Since the size of the training corpus affects the performance of an SRL system, we need to use corpora of the same size for training SMILE and BIOSMILE in order to accurately compare the effects of using newswire training data with those of using biomedical data. Because PropBank and BioProp are of different size, we use limited selections from both.

Before testing SMILE and BIOSMILE on BioProp, we train the two systems on different training sets of 30 randomly chosen sets from PropBank (*g*_1_,.., *g*_30_) and BioProp (*w*_1_,.., *w*_30_), respectively. Each set contains 1,000 PAS's. After the training process, we test both systems on 30 400-PAS test sets from BioProp (trained on *g*_1 _and *w*_1 _for use with test set 1, and trained on *g*_2 _and *w*_2 _for use with test set 2, etc.). We then sum the scores for *g*_1_-*g*_30 _and *w*_1_-*w*_30_, and calculate the averages for performance comparison. In Experiment 1, both SMILE and BIOSMILE use the baseline features illustrated in the Methods section. We denote the systems as SMILE and BIOSMILE_Baseline_, respectively.

### Experiment 2: the effect of using biomedical-specific features

To improve the performance of SRL on biomedical literature, we add two domain specific features, NE features and argument-template features (denoted as BIOSMILE_NE _and BIOSMILE_Template _respectively) to BIOSMILE. This experiment tests the effectiveness of adding the features to BIOSMILE_Baseline _and uses the same datasets as BIOSMILE_Baseline_.

Bio-specific NE features are created for each of the following five primary named entity (NE) categories in the GENIA ontology^3^: protein, nucleotide, other organic compounds, source, and others. When a constituent (node on the full-parsing tree) matches an NE exactly, the corresponding NE feature is enabled.

Additionally, we integrate argument-template features. Usually, each argument type has its own patterns. For example, in the biomedical domain, the regular expression "in * cell" is a locative argument pattern (ArgM-LOC). We automatically generate argument templates, which are composed of words, NEs, and POS's, to represent the patterns of each argument. These templates are generated by using the Smith and Waterman local alignment algorithm [35] to align all instances of a specific argument type. The template feature is enabled if a constituent matches a template exactly. NE features and argument template features are discussed further in the Methods section.

### Evaluation metrics

The results are given as F-scores using the CoNLL-05 evaluation script and defined as F = (2PR)/(P + R), where P denotes the precision and R denotes the recall. The formulas for calculating precision and recall are as follows:

Precision=the number of correctly recognized argumentsthe number of recognized argumentsRecall=the number of correctly recognized argumentsthe number of true arguments
 MathType@MTEF@5@5@+=feaafiart1ev1aaatCvAUfKttLearuWrP9MDH5MBPbIqV92AaeXatLxBI9gBaebbnrfifHhDYfgasaacH8akY=wiFfYdH8Gipec8Eeeu0xXdbba9frFj0=OqFfea0dXdd9vqai=hGuQ8kuc9pgc9s8qqaq=dirpe0xb9q8qiLsFr0=vr0=vr0dc8meaabaqaciaacaGaaeqabaqabeGadaaakqaaeeqaaiabbcfaqjabbkhaYjabbwgaLjabbogaJjabbMgaPjabbohaZjabbMgaPjabb+gaVjabb6gaUjabg2da9maalaaabaGaeeiDaqNaeeiAaGMaeeyzauMaeeiiaaIaeeOBa4MaeeyDauNaeeyBa0MaeeOyaiMaeeyzauMaeeOCaiNaeeiiaaIaee4Ba8MaeeOzayMaeeiiaaIaee4yamMaee4Ba8MaeeOCaiNaeeOCaiNaeeyzauMaee4yamMaeeiDaqNaeeiBaWMaeeyEaKNaeeiiaaIaeeOCaiNaeeyzauMaee4yamMaee4Ba8Maee4zaCMaeeOBa4MaeeyAaKMaeeOEaONaeeyzauMaeeizaqMaeeiiaaIaeeyyaeMaeeOCaiNaee4zaCMaeeyDauNaeeyBa0MaeeyzauMaeeOBa4MaeeiDaqNaee4CamhabaGaeeiDaqNaeeiAaGMaeeyzauMaeeiiaaIaeeOBa4MaeeyDauNaeeyBa0MaeeOyaiMaeeyzauMaeeOCaiNaeeiiaaIaee4Ba8MaeeOzayMaeeiiaaIaeeOCaiNaeeyzauMaee4yamMaee4Ba8Maee4zaCMaeeOBa4MaeeyAaKMaeeOEaONaeeyzauMaeeizaqMaeeiiaaIaeeyyaeMaeeOCaiNaee4zaCMaeeyDauNaeeyBa0MaeeyzauMaeeOBa4MaeeiDaqNaee4CamhaaaqaaiabbkfasjabbwgaLjabbogaJjabbggaHjabbYgaSjabbYgaSjabg2da9maalaaabaGaeeiDaqNaeeiAaGMaeeyzauMaeeiiaaIaeeOBa4MaeeyDauNaeeyBa0MaeeOyaiMaeeyzauMaeeOCaiNaeeiiaaIaee4Ba8MaeeOzayMaeeiiaaIaee4yamMaee4Ba8MaeeOCaiNaeeOCaiNaeeyzauMaee4yamMaeeiDaqNaeeiBaWMaeeyEaKNaeeiiaaIaeeOCaiNaeeyzauMaee4yamMaee4Ba8Maee4zaCMaeeOBa4MaeeyAaKMaeeOEaONaeeyzauMaeeizaqMaeeiiaaIaeeyyaeMaeeOCaiNaee4zaCMaeeyDauNaeeyBa0MaeeyzauMaeeOBa4MaeeiDaqNaee4CamhabaGaeeiDaqNaeeiAaGMaeeyzauMaeeiiaaIaeeOBa4MaeeyDauNaeeyBa0MaeeOyaiMaeeyzauMaeeOCaiNaeeiiaaIaee4Ba8MaeeOzayMaeeiiaaIaeeiDaqNaeeOCaiNaeeyDauNaeeyzauMaeeiiaaIaeeyyaeMaeeOCaiNaee4zaCMaeeyDauNaeeyBa0MaeeyzauMaeeOBa4MaeeiDaqNaee4Camhaaaaaaa@05E3@

## Results

Table 7 shows all the configurations and the summarized results. The latter are reported as the mean precision (P^
 MathType@MTEF@5@5@+=feaafiart1ev1aaatCvAUfKttLearuWrP9MDH5MBPbIqV92AaeXatLxBI9gBaebbnrfifHhDYfgasaacH8akY=wiFfYdH8Gipec8Eeeu0xXdbba9frFj0=OqFfea0dXdd9vqai=hGuQ8kuc9pgc9s8qqaq=dirpe0xb9q8qiLsFr0=vr0=vr0dc8meaabaqaciaacaGaaeqabaqabeGadaaakeaacuWGqbaugaqcaaaa@2DE5@), recall (R^
 MathType@MTEF@5@5@+=feaafiart1ev1aaatCvAUfKttLearuWrP9MDH5MBPbIqV92AaeXatLxBI9gBaebbnrfifHhDYfgasaacH8akY=wiFfYdH8Gipec8Eeeu0xXdbba9frFj0=OqFfea0dXdd9vqai=hGuQ8kuc9pgc9s8qqaq=dirpe0xb9q8qiLsFr0=vr0=vr0dc8meaabaqaciaacaGaaeqabaqabeGadaaakeaacuWGsbGugaqcaaaa@2DE9@), and F-score (F^
 MathType@MTEF@5@5@+=feaafiart1ev1aaatCvAUfKttLearuWrP9MDH5MBPbIqV92AaeXatLxBI9gBaebbnrfifHhDYfgasaacH8akY=wiFfYdH8Gipec8Eeeu0xXdbba9frFj0=OqFfea0dXdd9vqai=hGuQ8kuc9pgc9s8qqaq=dirpe0xb9q8qiLsFr0=vr0=vr0dc8meaabaqaciaacaGaaeqabaqabeGadaaakeaacuWGgbGrgaqcaaaa@2DD1@) of thirty datasets. We examine the detailed statistics of all the experiments in Tables 8, 9, and 10. In the tables, as well as P^
 MathType@MTEF@5@5@+=feaafiart1ev1aaatCvAUfKttLearuWrP9MDH5MBPbIqV92AaeXatLxBI9gBaebbnrfifHhDYfgasaacH8akY=wiFfYdH8Gipec8Eeeu0xXdbba9frFj0=OqFfea0dXdd9vqai=hGuQ8kuc9pgc9s8qqaq=dirpe0xb9q8qiLsFr0=vr0=vr0dc8meaabaqaciaacaGaaeqabaqabeGadaaakeaacuWGqbaugaqcaaaa@2DE5@, R^
 MathType@MTEF@5@5@+=feaafiart1ev1aaatCvAUfKttLearuWrP9MDH5MBPbIqV92AaeXatLxBI9gBaebbnrfifHhDYfgasaacH8akY=wiFfYdH8Gipec8Eeeu0xXdbba9frFj0=OqFfea0dXdd9vqai=hGuQ8kuc9pgc9s8qqaq=dirpe0xb9q8qiLsFr0=vr0=vr0dc8meaabaqaciaacaGaaeqabaqabeGadaaakeaacuWGsbGugaqcaaaa@2DE9@, and F^
 MathType@MTEF@5@5@+=feaafiart1ev1aaatCvAUfKttLearuWrP9MDH5MBPbIqV92AaeXatLxBI9gBaebbnrfifHhDYfgasaacH8akY=wiFfYdH8Gipec8Eeeu0xXdbba9frFj0=OqFfea0dXdd9vqai=hGuQ8kuc9pgc9s8qqaq=dirpe0xb9q8qiLsFr0=vr0=vr0dc8meaabaqaciaacaGaaeqabaqabeGadaaakeaacuWGgbGrgaqcaaaa@2DD1@, we also list the sample standard deviation of the F-score (S^F
 MathType@MTEF@5@5@+=feaafiart1ev1aaatCvAUfKttLearuWrP9MDH5MBPbIqV92AaeXatLxBI9gBaebbnrfifHhDYfgasaacH8akY=wiFfYdH8Gipec8Eeeu0xXdbba9frFj0=OqFfea0dXdd9vqai=hGuQ8kuc9pgc9s8qqaq=dirpe0xb9q8qiLsFr0=vr0=vr0dc8meaabaqaciaacaGaaeqabaqabeGadaaakeaacuWGtbWugaqcamaaBaaaleaacqWGgbGraeqaaaaa@2F2C@) for each argument type. We apply a two-sample *t *test to examine whether one configuration is better than the other with statistical significance. The null hypothesis, which states that there is no difference between the two configurations, is given by

**Table 7:** Results of all configurations

**System**	**Training**	**Test**	**P^ MathType@MTEF@5@5@+=feaafiart1ev1aaatCvAUfKttLearuWrP9MDH5MBPbIqV92AaeXatLxBI9gBaebbnrfifHhDYfgasaacH8akY=wiFfYdH8Gipec8Eeeu0xXdbba9frFj0=OqFfea0dXdd9vqai=hGuQ8kuc9pgc9s8qqaq=dirpe0xb9q8qiLsFr0=vr0=vr0dc8meaabaqaciaacaGaaeqabaqabeGadaaakeaacuWGqbaugaqcaaaa@2DE5@ (%)**	**R^ MathType@MTEF@5@5@+=feaafiart1ev1aaatCvAUfKttLearuWrP9MDH5MBPbIqV92AaeXatLxBI9gBaebbnrfifHhDYfgasaacH8akY=wiFfYdH8Gipec8Eeeu0xXdbba9frFj0=OqFfea0dXdd9vqai=hGuQ8kuc9pgc9s8qqaq=dirpe0xb9q8qiLsFr0=vr0=vr0dc8meaabaqaciaacaGaaeqabaqabeGadaaakeaacuWGsbGugaqcaaaa@2DE9@ (%)**	**F^ MathType@MTEF@5@5@+=feaafiart1ev1aaatCvAUfKttLearuWrP9MDH5MBPbIqV92AaeXatLxBI9gBaebbnrfifHhDYfgasaacH8akY=wiFfYdH8Gipec8Eeeu0xXdbba9frFj0=OqFfea0dXdd9vqai=hGuQ8kuc9pgc9s8qqaq=dirpe0xb9q8qiLsFr0=vr0=vr0dc8meaabaqaciaacaGaaeqabaqabeGadaaakeaacuWGgbGrgaqcaaaa@2DD1@ (%)**
SMILE	PropBank I	BioProp	74.95	54.05	62.80
BIOSMILE_Baseline_	BioProp	BioProp	87.03	81.65	84.25
BIOSMILE_NE_	BioProp	BioProp	87.31	81.66	84.38
BIOSMILE_Template_	BioProp	BioProp	87.56	82.15	84.76

**Table 8:** Comparison of performance on SMILE and BIOSMILE_Baseline_

	SMILE				BIOSMILE_Baseline_						
				
Type	P^ MathType@MTEF@5@5@+=feaafiart1ev1aaatCvAUfKttLearuWrP9MDH5MBPbIqV92AaeXatLxBI9gBaebbnrfifHhDYfgasaacH8akY=wiFfYdH8Gipec8Eeeu0xXdbba9frFj0=OqFfea0dXdd9vqai=hGuQ8kuc9pgc9s8qqaq=dirpe0xb9q8qiLsFr0=vr0=vr0dc8meaabaqaciaacaGaaeqabaqabeGadaaakeaacuWGqbaugaqcaaaa@2DE5@ (%)	R^ MathType@MTEF@5@5@+=feaafiart1ev1aaatCvAUfKttLearuWrP9MDH5MBPbIqV92AaeXatLxBI9gBaebbnrfifHhDYfgasaacH8akY=wiFfYdH8Gipec8Eeeu0xXdbba9frFj0=OqFfea0dXdd9vqai=hGuQ8kuc9pgc9s8qqaq=dirpe0xb9q8qiLsFr0=vr0=vr0dc8meaabaqaciaacaGaaeqabaqabeGadaaakeaacuWGsbGugaqcaaaa@2DE9@ (%)	F^ MathType@MTEF@5@5@+=feaafiart1ev1aaatCvAUfKttLearuWrP9MDH5MBPbIqV92AaeXatLxBI9gBaebbnrfifHhDYfgasaacH8akY=wiFfYdH8Gipec8Eeeu0xXdbba9frFj0=OqFfea0dXdd9vqai=hGuQ8kuc9pgc9s8qqaq=dirpe0xb9q8qiLsFr0=vr0=vr0dc8meaabaqaciaacaGaaeqabaqabeGadaaakeaacuWGgbGrgaqcaaaa@2DD1@ (%)	S^F MathType@MTEF@5@5@+=feaafiart1ev1aaatCvAUfKttLearuWrP9MDH5MBPbIqV92AaeXatLxBI9gBaebbnrfifHhDYfgasaacH8akY=wiFfYdH8Gipec8Eeeu0xXdbba9frFj0=OqFfea0dXdd9vqai=hGuQ8kuc9pgc9s8qqaq=dirpe0xb9q8qiLsFr0=vr0=vr0dc8meaabaqaciaacaGaaeqabaqabeGadaaakeaacuWGtbWugaqcamaaBaaaleaacqWGgbGraeqaaaaa@2F2C@ (%)	P^ MathType@MTEF@5@5@+=feaafiart1ev1aaatCvAUfKttLearuWrP9MDH5MBPbIqV92AaeXatLxBI9gBaebbnrfifHhDYfgasaacH8akY=wiFfYdH8Gipec8Eeeu0xXdbba9frFj0=OqFfea0dXdd9vqai=hGuQ8kuc9pgc9s8qqaq=dirpe0xb9q8qiLsFr0=vr0=vr0dc8meaabaqaciaacaGaaeqabaqabeGadaaakeaacuWGqbaugaqcaaaa@2DE5@ (%)	R^ MathType@MTEF@5@5@+=feaafiart1ev1aaatCvAUfKttLearuWrP9MDH5MBPbIqV92AaeXatLxBI9gBaebbnrfifHhDYfgasaacH8akY=wiFfYdH8Gipec8Eeeu0xXdbba9frFj0=OqFfea0dXdd9vqai=hGuQ8kuc9pgc9s8qqaq=dirpe0xb9q8qiLsFr0=vr0=vr0dc8meaabaqaciaacaGaaeqabaqabeGadaaakeaacuWGsbGugaqcaaaa@2DE9@ (%)	F^ MathType@MTEF@5@5@+=feaafiart1ev1aaatCvAUfKttLearuWrP9MDH5MBPbIqV92AaeXatLxBI9gBaebbnrfifHhDYfgasaacH8akY=wiFfYdH8Gipec8Eeeu0xXdbba9frFj0=OqFfea0dXdd9vqai=hGuQ8kuc9pgc9s8qqaq=dirpe0xb9q8qiLsFr0=vr0=vr0dc8meaabaqaciaacaGaaeqabaqabeGadaaakeaacuWGgbGrgaqcaaaa@2DD1@ (%)	S^F MathType@MTEF@5@5@+=feaafiart1ev1aaatCvAUfKttLearuWrP9MDH5MBPbIqV92AaeXatLxBI9gBaebbnrfifHhDYfgasaacH8akY=wiFfYdH8Gipec8Eeeu0xXdbba9frFj0=OqFfea0dXdd9vqai=hGuQ8kuc9pgc9s8qqaq=dirpe0xb9q8qiLsFr0=vr0=vr0dc8meaabaqaciaacaGaaeqabaqabeGadaaakeaacuWGtbWugaqcamaaBaaaleaacqWGgbGraeqaaaaa@2F2C@ (%)	ΔF (%)	*t*	*F*_*B*_>*F*_*S*_? (*t *>1.96?)
Arg0	85.66	63.47	72.86	2.66	92.33	90.52	91.41	1.44	18.55	33.59	Y
Arg1	82.10	75.02	78.39	1.96	88.86	85.71	87.25	1.42	8.86	20.05	Y
Arg2	39.58	30.69	34.35	5.73	86.46	81.26	83.68	3.93	49.33	38.89	Y
ArgM-ADV	38.59	22.52	27.94	7.96	64.14	51.20	56.60	5.77	28.66	15.97	Y
ArgM-DIS	72.58	52.12	59.92	8.62	83.74	74.91	78.83	5.39	18.91	10.19	Y
ArgM-LOC	62.17	1.98	3.79	3.60	76.03	77.12	76.48	3.67	72.69	77.45	Y
ArgM-MNR	45.29	18.61	25.95	6.99	83.30	81.02	82.04	2.74	56.09	40.92	Y
ArgM-MOD	99.25	87.48	92.84	3.66	97.22	94.67	95.82	2.36	2.98	3.75	Y
ArgM-NEG	99.37	76.77	86.24	6.66	97.70	94.98	96.17	2.80	9.93	7.53	Y
ArgM-TMP	71.60	57.33	62.98	9.88	81.48	61.65	69.67	7.25	6.69	2.99	Y

**Overall**	**74.95 **	**54.05 **	**62.80 **	**1.95 **	**87.03 **	**81.65 **	**84.25 **	**1.33 **	**21.45 **	**49.82 **	**Y**

**Table 9:** Comparison of performance on BIOSMILE_Baseline _and BIOSMILE_NE_

	BIOSMILE_Baseline_				BIOSMILE_NE_						
				
Type	P^ MathType@MTEF@5@5@+=feaafiart1ev1aaatCvAUfKttLearuWrP9MDH5MBPbIqV92AaeXatLxBI9gBaebbnrfifHhDYfgasaacH8akY=wiFfYdH8Gipec8Eeeu0xXdbba9frFj0=OqFfea0dXdd9vqai=hGuQ8kuc9pgc9s8qqaq=dirpe0xb9q8qiLsFr0=vr0=vr0dc8meaabaqaciaacaGaaeqabaqabeGadaaakeaacuWGqbaugaqcaaaa@2DE5@ (%)	R^ MathType@MTEF@5@5@+=feaafiart1ev1aaatCvAUfKttLearuWrP9MDH5MBPbIqV92AaeXatLxBI9gBaebbnrfifHhDYfgasaacH8akY=wiFfYdH8Gipec8Eeeu0xXdbba9frFj0=OqFfea0dXdd9vqai=hGuQ8kuc9pgc9s8qqaq=dirpe0xb9q8qiLsFr0=vr0=vr0dc8meaabaqaciaacaGaaeqabaqabeGadaaakeaacuWGsbGugaqcaaaa@2DE9@ (%)	F^ MathType@MTEF@5@5@+=feaafiart1ev1aaatCvAUfKttLearuWrP9MDH5MBPbIqV92AaeXatLxBI9gBaebbnrfifHhDYfgasaacH8akY=wiFfYdH8Gipec8Eeeu0xXdbba9frFj0=OqFfea0dXdd9vqai=hGuQ8kuc9pgc9s8qqaq=dirpe0xb9q8qiLsFr0=vr0=vr0dc8meaabaqaciaacaGaaeqabaqabeGadaaakeaacuWGgbGrgaqcaaaa@2DD1@ (%)	S^F MathType@MTEF@5@5@+=feaafiart1ev1aaatCvAUfKttLearuWrP9MDH5MBPbIqV92AaeXatLxBI9gBaebbnrfifHhDYfgasaacH8akY=wiFfYdH8Gipec8Eeeu0xXdbba9frFj0=OqFfea0dXdd9vqai=hGuQ8kuc9pgc9s8qqaq=dirpe0xb9q8qiLsFr0=vr0=vr0dc8meaabaqaciaacaGaaeqabaqabeGadaaakeaacuWGtbWugaqcamaaBaaaleaacqWGgbGraeqaaaaa@2F2C@ (%)	P^ MathType@MTEF@5@5@+=feaafiart1ev1aaatCvAUfKttLearuWrP9MDH5MBPbIqV92AaeXatLxBI9gBaebbnrfifHhDYfgasaacH8akY=wiFfYdH8Gipec8Eeeu0xXdbba9frFj0=OqFfea0dXdd9vqai=hGuQ8kuc9pgc9s8qqaq=dirpe0xb9q8qiLsFr0=vr0=vr0dc8meaabaqaciaacaGaaeqabaqabeGadaaakeaacuWGqbaugaqcaaaa@2DE5@ (%)	R^ MathType@MTEF@5@5@+=feaafiart1ev1aaatCvAUfKttLearuWrP9MDH5MBPbIqV92AaeXatLxBI9gBaebbnrfifHhDYfgasaacH8akY=wiFfYdH8Gipec8Eeeu0xXdbba9frFj0=OqFfea0dXdd9vqai=hGuQ8kuc9pgc9s8qqaq=dirpe0xb9q8qiLsFr0=vr0=vr0dc8meaabaqaciaacaGaaeqabaqabeGadaaakeaacuWGsbGugaqcaaaa@2DE9@ (%)	F^ MathType@MTEF@5@5@+=feaafiart1ev1aaatCvAUfKttLearuWrP9MDH5MBPbIqV92AaeXatLxBI9gBaebbnrfifHhDYfgasaacH8akY=wiFfYdH8Gipec8Eeeu0xXdbba9frFj0=OqFfea0dXdd9vqai=hGuQ8kuc9pgc9s8qqaq=dirpe0xb9q8qiLsFr0=vr0=vr0dc8meaabaqaciaacaGaaeqabaqabeGadaaakeaacuWGgbGrgaqcaaaa@2DD1@ (%)	S^F MathType@MTEF@5@5@+=feaafiart1ev1aaatCvAUfKttLearuWrP9MDH5MBPbIqV92AaeXatLxBI9gBaebbnrfifHhDYfgasaacH8akY=wiFfYdH8Gipec8Eeeu0xXdbba9frFj0=OqFfea0dXdd9vqai=hGuQ8kuc9pgc9s8qqaq=dirpe0xb9q8qiLsFr0=vr0=vr0dc8meaabaqaciaacaGaaeqabaqabeGadaaakeaacuWGtbWugaqcamaaBaaaleaacqWGgbGraeqaaaaa@2F2C@ (%)	ΔF (%)	*t*	*F*_*N*_>*F*_*B*_? (*t *>1.67?)
Arg0	92.33	90.52	91.41	1.44	92.29	90.46	91.35	1.53	-0.05	-0.14	N
Arg1	88.86	85.71	87.25	1.42	89.32	86.07	87.66	1.31	0.41	1.18	N
Arg2	86.46	81.26	83.68	3.93	86.78	81.07	83.73	4.39	0.05	0.05	N
ArgM-ADV	64.14	51.20	56.60	5.77	64.73	50.90	56.61	6.06	0.01	0.01	N
ArgM-DIS	83.74	74.91	78.83	5.39	84.14	74.71	78.81	5.66	-0.02	-0.01	N
ArgM-LOC	76.03	77.12	76.48	3.67	76.54	77.06	76.71	3.74	0.23	0.25	N
ArgM-MNR	83.30	81.02	82.04	2.74	83.05	81.20	82.02	2.79	-0.02	-0.03	N
ArgM-MOD	97.22	94.67	95.82	2.36	97.31	94.47	95.76	2.68	-0.05	-0.08	N
ArgM-NEG	97.70	94.98	96.17	2.80	97.45	94.97	96.03	2.91	-0.14	-0.19	N
ArgM-TMP	81.48	61.65	69.67	7.25	81.80	61.33	69.62	7.31	-0.05	-0.03	N

**Overall**	**87.03 **	**81.65 **	**84.25 **	**1.33 **	**87.31 **	**81.66 **	**84.38 **	**1.37 **	**0.14 **	**0.40 **	**N**

**Table 10:** Comparison of performance on BIOSMILE_Baseline _and BIOSMILE_Template_

	BIOSMILE_Baseline_				BIOSMILE_Template_						
				
Type	P^ MathType@MTEF@5@5@+=feaafiart1ev1aaatCvAUfKttLearuWrP9MDH5MBPbIqV92AaeXatLxBI9gBaebbnrfifHhDYfgasaacH8akY=wiFfYdH8Gipec8Eeeu0xXdbba9frFj0=OqFfea0dXdd9vqai=hGuQ8kuc9pgc9s8qqaq=dirpe0xb9q8qiLsFr0=vr0=vr0dc8meaabaqaciaacaGaaeqabaqabeGadaaakeaacuWGqbaugaqcaaaa@2DE5@ (%)	R^ MathType@MTEF@5@5@+=feaafiart1ev1aaatCvAUfKttLearuWrP9MDH5MBPbIqV92AaeXatLxBI9gBaebbnrfifHhDYfgasaacH8akY=wiFfYdH8Gipec8Eeeu0xXdbba9frFj0=OqFfea0dXdd9vqai=hGuQ8kuc9pgc9s8qqaq=dirpe0xb9q8qiLsFr0=vr0=vr0dc8meaabaqaciaacaGaaeqabaqabeGadaaakeaacuWGsbGugaqcaaaa@2DE9@ (%)	F^ MathType@MTEF@5@5@+=feaafiart1ev1aaatCvAUfKttLearuWrP9MDH5MBPbIqV92AaeXatLxBI9gBaebbnrfifHhDYfgasaacH8akY=wiFfYdH8Gipec8Eeeu0xXdbba9frFj0=OqFfea0dXdd9vqai=hGuQ8kuc9pgc9s8qqaq=dirpe0xb9q8qiLsFr0=vr0=vr0dc8meaabaqaciaacaGaaeqabaqabeGadaaakeaacuWGgbGrgaqcaaaa@2DD1@ (%)	S^F MathType@MTEF@5@5@+=feaafiart1ev1aaatCvAUfKttLearuWrP9MDH5MBPbIqV92AaeXatLxBI9gBaebbnrfifHhDYfgasaacH8akY=wiFfYdH8Gipec8Eeeu0xXdbba9frFj0=OqFfea0dXdd9vqai=hGuQ8kuc9pgc9s8qqaq=dirpe0xb9q8qiLsFr0=vr0=vr0dc8meaabaqaciaacaGaaeqabaqabeGadaaakeaacuWGtbWugaqcamaaBaaaleaacqWGgbGraeqaaaaa@2F2C@ (%)	P^ MathType@MTEF@5@5@+=feaafiart1ev1aaatCvAUfKttLearuWrP9MDH5MBPbIqV92AaeXatLxBI9gBaebbnrfifHhDYfgasaacH8akY=wiFfYdH8Gipec8Eeeu0xXdbba9frFj0=OqFfea0dXdd9vqai=hGuQ8kuc9pgc9s8qqaq=dirpe0xb9q8qiLsFr0=vr0=vr0dc8meaabaqaciaacaGaaeqabaqabeGadaaakeaacuWGqbaugaqcaaaa@2DE5@ (%)	R^ MathType@MTEF@5@5@+=feaafiart1ev1aaatCvAUfKttLearuWrP9MDH5MBPbIqV92AaeXatLxBI9gBaebbnrfifHhDYfgasaacH8akY=wiFfYdH8Gipec8Eeeu0xXdbba9frFj0=OqFfea0dXdd9vqai=hGuQ8kuc9pgc9s8qqaq=dirpe0xb9q8qiLsFr0=vr0=vr0dc8meaabaqaciaacaGaaeqabaqabeGadaaakeaacuWGsbGugaqcaaaa@2DE9@ (%)	F^ MathType@MTEF@5@5@+=feaafiart1ev1aaatCvAUfKttLearuWrP9MDH5MBPbIqV92AaeXatLxBI9gBaebbnrfifHhDYfgasaacH8akY=wiFfYdH8Gipec8Eeeu0xXdbba9frFj0=OqFfea0dXdd9vqai=hGuQ8kuc9pgc9s8qqaq=dirpe0xb9q8qiLsFr0=vr0=vr0dc8meaabaqaciaacaGaaeqabaqabeGadaaakeaacuWGgbGrgaqcaaaa@2DD1@ (%)	S^F MathType@MTEF@5@5@+=feaafiart1ev1aaatCvAUfKttLearuWrP9MDH5MBPbIqV92AaeXatLxBI9gBaebbnrfifHhDYfgasaacH8akY=wiFfYdH8Gipec8Eeeu0xXdbba9frFj0=OqFfea0dXdd9vqai=hGuQ8kuc9pgc9s8qqaq=dirpe0xb9q8qiLsFr0=vr0=vr0dc8meaabaqaciaacaGaaeqabaqabeGadaaakeaacuWGtbWugaqcamaaBaaaleaacqWGgbGraeqaaaaa@2F2C@ (%)	ΔF (%)	*t*	*F*_*T*_>*F*_*B*_? (*t *>1.67?)
Arg0	92.33	90.52	91.41	1.44	92.35	90.48	91.40	1.52	-0.01	-0.02	N
Arg1	88.86	85.71	87.25	1.42	88.83	85.75	87.25	1.39	0.00	0.01	N
Arg2	86.46	81.26	83.68	3.93	86.45	81.63	83.87	3.93	0.19	0.19	N
ArgM-ADV	64.14	51.20	56.60	5.77	66.96	54.77	59.93	5.83	3.33	2.22	Y
ArgM-DIS	83.74	74.91	78.83	5.39	84.14	74.92	78.99	5.39	0.16	0.12	N
ArgM-LOC	76.03	77.12	76.48	3.67	79.65	78.07	78.75	3.21	2.27	2.55	Y
ArgM-MNR	83.30	81.02	82.04	2.74	84.15	83.02	83.49	2.69	1.44	2.06	Y
ArgM-MOD	97.22	94.67	95.82	2.36	97.55	94.67	96.00	2.39	0.18	0.29	N
ArgM-NEG	97.70	94.98	96.17	2.80	97.70	94.98	96.17	2.80	0.00	0.00	N
ArgM-TMP	81.48	61.65	69.67	7.25	83.90	63.33	71.75	6.32	2.08	1.18	N

**Overall**	**87.03 **	**81.65 **	**84.25 **	**1.33 **	**87.56 **	**82.15 **	**84.76 **	**1.35 **	**0.52 **	**1.50 **	**N**

H_0_: *μ*_A _= *μ*_B_,

where *μ*_A _is the true mean F-score of configuration A, *μ*_B _is the mean of configuration B, and the alternative hypothesis is

H_1_: *μ*_A _> *μ*_B_.

A two-sample *t-*test is applied since we assume the samples are independent. As the number of samples is large and the samples' standard deviations are known, the following two-sample *t*-statistic is appropriate in this case:

t=(x¯A−x¯B)SA2nA+SB2nB.
 MathType@MTEF@5@5@+=feaafiart1ev1aaatCvAUfKttLearuWrP9MDH5MBPbIqV92AaeXatLxBI9gBaebbnrfifHhDYfgasaacH8akY=wiFfYdH8Gipec8Eeeu0xXdbba9frFj0=OqFfea0dXdd9vqai=hGuQ8kuc9pgc9s8qqaq=dirpe0xb9q8qiLsFr0=vr0=vr0dc8meaabaqaciaacaGaaeqabaqabeGadaaakeaacqWG0baDcqGH9aqpdaWcaaqaaiabcIcaOiqbdIha4zaaraWaaSbaaSqaaiabdgeabbqabaGccqGHsislcuWG4baEgaqeamaaBaaaleaacqWGcbGqaeqaaOGaeiykaKcabaWaaOaaaeaadaWcaaqaaiabdofatnaaDaaaleaacqWGbbqqaeaacqaIYaGmaaaakeaacqWGUbGBdaWgaaWcbaGaemyqaeeabeaaaaGccqGHRaWkdaWcaaqaaiabdofatnaaDaaaleaacqWGcbGqaeaacqaIYaGmaaaakeaacqWGUbGBdaWgaaWcbaGaemOqaieabeaaaaaabeaaaaGccqGGUaGlaaa@4584@

If the resulting *t *score is equal to or less than 1.67 with a degree of freedom of 29 and a statistical significance level of 95%, the null hypothesis is accepted; otherwise it is rejected. Even though we do not list all the argument types in Tables 8, 9, and 10 (because some argument types only have a few instances), we calculate the overall score by checking all argument types.

### Experiment 1

Table 8 shows the results of Experiment 1. We find that BIOSMILE outperforms SMILE by 21.45% on the F-score when tested on BioProp. It also outperforms SMILE for all argument types with a statistically significant difference. Specifically, ArgM-LOC, ArgM-MNR, and Arg2 achieve the biggest performance improvement, followed by ArgM-ADV, ArgM-DIS, and Arg0.

### Experiment 2

Table 9 compares the performance of BIOSMILE_Baseline _and BIOSMILE_NE_. Initially, we expected that the NE features would improve the recognition of adjunct arguments (ArgM), such as ArgM-LOC. However, they failed to do so.

**Figure 3:**
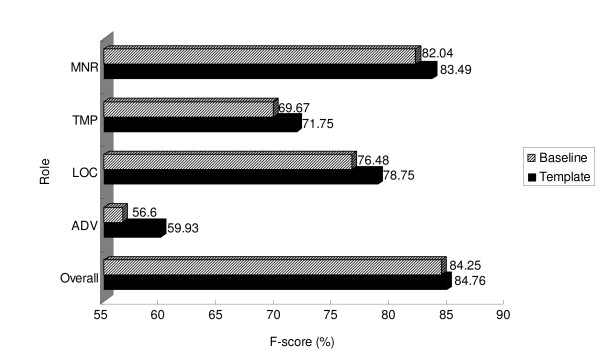
Performance improvement of template features overall and on several adjunct argument types.

Argument template features, on the other hand, boost the system's performance. Table 10 compares the performance of argument templates on BIOSMILE_Baseline _and BIOSMILE_Template_. The overall F-score is only improved slightly (0.52%). However, we achieve 3.33%, 2.27%, and 1.44% increases in the F-scores of ArgM-ADV, ArgM-LOC, and ArgM-MNR, respectively. These improvements are statistically significant. Although the increase in ArgM-TMP's F-score is not statistically significant, it is still appreciable. Figure 3 gives a clearer illustration of the improved performance of these argument types.

## Discussion

Experiment 1 demonstrates that BIOSMILE_Baseline _outperforms SMILE by more than 20%. Experiment 2 shows that NE features do not improve BIOSMILE's performance, but template features do. Next, we further analyze the results and discuss possible reasons for them.

### The influence of sense change on the biomedical and newswire domains

According to the results of Experiment 1, verbs with different framesets in the newswire and biomedical domain exhibit larger differences in performance between SMILE and BIOSMILE_Baseline_. Table 11 details the average F-score difference between verbs with different framesets in both domains and verbs with the same framesets in both domains; the verb types are defined in the Background section (Verb Selection). These performance differences suggest that variations in cross-domain framesets and the usage of specific verbs influence SRL performance.

**Table 11:** Comparison of performance difference on verbs that have different framesets and the same framesets in Experiment 1

**Verb Type**	FBIOSMILEBaseline MathType@MTEF@5@5@+=feaafiart1ev1aaatCvAUfKttLearuWrP9MDH5MBPbIqV92AaeXatLxBI9gBaebbnrfifHhDYfgasaacH8akY=wiFfYdH8Gipec8Eeeu0xXdbba9frFj0=OqFfea0dXdd9vqai=hGuQ8kuc9pgc9s8qqaq=dirpe0xb9q8qiLsFr0=vr0=vr0dc8meaabaqaciaacaGaaeqabaqabeGadaaakeaaieqacqWFgbGrdaWgaaWcbaGae8NqaiKae8xsaKKae83ta8Kae83uamLae8xta0Kae8xsaKKae8htaWKae8xrau0aaSbaaWqaaiab=jeacjab=fgaHjab=nhaZjab=vgaLjab=XgaSjab=LgaPjab=5gaUjab=vgaLbqabaaaleqaaaaa@4169@ - **F**_SMILE_
Different frame set	28.12%
The same set	18.42%

### Why NE features are not effective

In the newswire domain, NE features have proven effective in improving SRL performance. However, the results of Experiment 2 show that we did not have the same success in the biomedical domain. Table 12 lists the five NE classes used and the number of NEs that occur in the main arguments. Though arguments frequently contain NEs, according to our findings, the converse does not hold. We find that most NEs match the NULL arguments, i.e., they match the nodes that are not labelled by BIOSMILE and, equivalently, do not correspond to the argument types of interest to us. Thus, trained on this data, a machine learning model would give so much weight to the NULL class that it would render all NE features ineffective for argument classification.

**Table 12:** Distribution of NEs in the main and NULL arguments

	**A0**	**A1**	**A2**	**NULL**
Compound	97	7	0	2361
Space	46	48	0	5371
Protein	167	169	13	10651
Other	25	260	0	4860
Nucleotide	18	36	1	3753

### Performance gained using template features

Only five argument types have a sufficient number of generated templates to be useful: ArgM-DIS, ArgM-MNR, ArgM-ADV, ArgM-TMP, and ArgM-LOC. We do not generate templates for arguments like ArgM-MOD and ArgM-NEG as they are usually composed of single words, such as "can" (ArgM-MOD) and "not" (ArgM-NEG). Baseline features, such as headword features, can generally recognize these arguments with a high degree of accuracy (F-scores of 95.82% for ArgM-MOD and 96.17% for ArgM-NEG). Templates for Arg0 and Arg1 are difficult to implement because a phrase pattern tagged as Arg0 may be tagged as Arg1 elsewhere, which results in all generated patterns being filtered out. Table 13 lists the performance improvement generated by template features on the five specified argument types, based on the improvement of the F-score over the baseline BIOSMILE. It also reports the number of templates, the number of instances, and the template density (# of templates/# of instances) for each type.

**Table 13:** Template feature statistics for the five argument types

	F-score (%)					
					
Argument Type	Baseline	Template	ΔF (%)	# of templates	# of instances	Template Density
ArgM-ADV	56.60	59.93	3.33	88	301	0.292359
ArgM-DIS	78.83	78.99	0.16	2	22	0.090909
ArgM-LOC	76.48	78.75	2.27	274	377	0.726790
ArgM-MNR	82.04	83.49	1.44	72	477	0.150943
ArgM-TMP	69.67	71.75	2.08	57	141	0.404255

**Figure 4:**
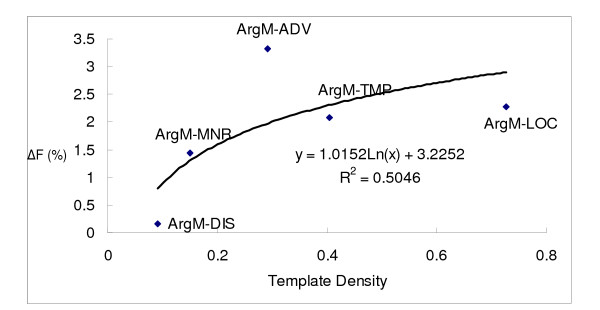
Relationship betweenΔF and template density.

**Figure 5:**
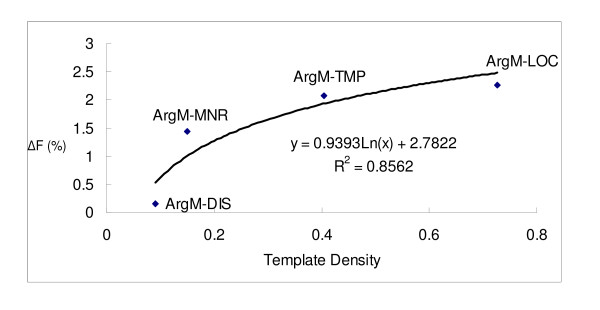
Relationship betweenΔF and template density after removing ArgM-ADV.

Figures 4 and 5 further illustrate the positive logarithmic correlation between template density and F-score difference (ΔF) for each argument type. ΔF initially increases with template density, but then appears to taper off. Note that the R-squared between ΔF and the logarithmic template density is 0.5046. However, after removing ArgM-ADV from the data, the R-squared increases to 0.8562, as shown in Figure 5. There are two possible explanations for ArgM-ADV's exceptionally high ΔF. First, its baseline F-score value is the lowest (56.60%) among the five adjunct arguments. Thus, increasing its F-score would be the easiest among the five arguments. Second, its average length is the longest so that its templates longer and possibly more precise.

Here, we give an example to illustrate how template features recognize an ArgM-TMP. Table 14 shows the features of each word in the sentence: "NAC not only blocks the effect of TPCK, but also enhances mitogenesis and cytokine production (>2.5-fold in some cases) upon activation of unsuppressed T cells." The phrase "upon activation of unsuppressed T cells" matches the ArgM-TMP template "*IN NN IN SRC SRC *cells". Each template slot indicates the allowable real words, NE tags, or POS tags. (Please refer to the Named Entity Features section to find all the abbreviations of NE tags.) However, the baseline BIOSMILE can not recognize this phrase by itself (shown in column five). Turning template features on, however, correctly identifies the ArgM-TMP, as shown in column six.

**Table 14:** An example of using an ArgM-TMP template

**Words**	**NE**	**POS**	**Predicate**	**BIOSMILE**_Baseline_	**BIOSMILE**_Template_
NAC	(PTN*)	NN	-	(Arg0*)	(Arg0*)
Not	*	RB	-	*	*
Only	*	RB	-	*	*
blocks	*	VBZ	-	*	*
The	*	DT	-	*	*
Effect	*	NN	-	*	*
Of	*	IN	-	*	*
TPCK	(OOC*)	NN	-	*	*
But	*	CC	-	*	*
enhances	*	VBZ	enhance	(V*)	(V*)
mitogenesis	*	NN	-	(Arg1*	(Arg1*
And	*	CC	-	*	*
cytokine	(OTR(PTN*)	NN	-	*	*
production	*)	NN	-	*)	*)
(	*	-LRB-	-	(ArgM-EXT*	(ArgM-EXT*
>	*	JJR	-	*	*
2.5-fold	*	RB	-	*	*
In	*	IN	-	*	*
some	*	DT	-	*	*
cases	*	NNS	-	*	*
)	*	-RRB-	-	*)	*)
upon	*	IN	-	*	(ArgM-TMP*
activation	*	NN	-	*	*
of	*	IN	-	*	*
unsuppressed	(SRC*	JJ	-	*	*
T	(SRC*	NN	-	*	*
cells	*))	NNS	-	*	*)
	*		-	*	*

## Conclusion

To improve the performance of SRL in the biomedical domain, we have developed BIOSMILE, a biomedical SRL system trained on a biomedical proposition bank called BioProp. The construction of BioProp is based on a semi-automatic strategy. Since our experiment results show that the differences in framesets and the usage variations of verbs in the biomedical and newswire proposition banks affect the performance of the underlying SRL systems, the necessity of training BIOSMILE on a biomedical proposition bank has been demonstrated. BIOSMILE is capable of processing the PAS' of thirty verbs selected according to their frequency and importance in describing molecular events. Incorporating automatically generated templates enhances the overall performance of argument classification, especially for locations, manners, and adverbs.

Finally, the following related issues remain to be addressed in our future work: (1) enhancing the system by adding more biomedical verbs to BioProp and integrating an automatic Penn-style parser into BIOSMILE; (2) applying BIOSMILE to other biomedical text mining systems, such as relation extraction and question answering systems; (3) examining the effectiveness of using BIOSMILE in other biomedical corpora; and (4) extracting biomedical relations expressed across sentences through SRL.

## Methods

First, we briefly introduce the machine learning model and features used in SMILE and BIOSMILE. Then, we explain the specific biomedical features of BIOSMILE, which we discussed in the Results and Discussion sections, in more detail.

### Formulation of semantic role labeling

Like POS tagging, chunking, and named entity recognition, SRL can be formulated as a sentence tagging problem. A sentence can be represented by a sequence of words, a sequence of phrases, or a parsing tree; the basic units of a sentence are words, phrases, and constituents (a node on a full parsing tree) arranged in the above representations, respectively. Hacioglu et al. [36] showed that tagging phrase-by-phrase (P-by-P) is better than word-by-word (W-by-W). However, Punyakanok et al. [15] showed that constituent-by-constituent (C-by-C, or node-by-node) tagging is better than P-by-P. Therefore, we adopt C-by-C tagging for SRL.

In the following subsections, we first describe the maximum entropy model used for argument classification, and then illustrate the basic features of our SMILE and BIOSMILE systems.

### Maximum entropy model

The maximum entropy (ME) model is a flexible statistical framework that assigns an outcome for each instance based on the instance's history, which is made up of all the conditioning data that enables one to assign probabilities to the space of all outcomes. In SRL, a history can be viewed as all the information related to the current token that is derivable from the training corpus. ME computes the probability, *p*(*o*|*h*), for any *o *from the space of all possible outcomes, *O*, and for every *h *from the space of all possible histories, *H*.

The computation of *p*(*o*|*h*) in an ME depends on a set of binary features, which are useful for making predictions about the outcome. For instance, a node in the parsing tree that ends with "cell" is very likely to be an ArgM-LOC. Formally, we can represent this feature as follows:

f(h,o)={1:ifcurrent_node_ends_with_cell(h)=trueand o=ArgM-LOC0:otherwise.
 MathType@MTEF@5@5@+=feaafiart1ev1aaatCvAUfKttLearuWrP9MDH5MBPbIqV92AaeXatLxBI9gBaebbnrfifHhDYfgasaacH8akY=wiFfYdH8Gipec8Eeeu0xXdbba9frFj0=OqFfea0dXdd9vqai=hGuQ8kuc9pgc9s8qqaq=dirpe0xb9q8qiLsFr0=vr0=vr0dc8meaabaqaciaacaGaaeqabaqabeGadaaakeaacqWGMbGzcqGGOaakcqWGObaAcqGGSaalcqWGVbWBcqGGPaqkcqGH9aqpdaGabeqaauaabaqadiaaaeaacqaIXaqmcqGG6aGoaeaacqqGPbqAcqqGMbGzcqGGGaaicqGGJbWycqGG1bqDcqGGYbGCcqGGYbGCcqGGLbqzcqGGUbGBcqGG0baDcqGGFbWxcqGGUbGBcqGGVbWBcqGGKbazcqGGLbqzcqGGFbWxcqGGLbqzcqGGUbGBcqGGKbazcqGGZbWCcqGGFbWxcqWG3bWDcqWGPbqAcqWG0baDcqWGObaAcqGGFbWxcqGGJbWycqGGLbqzcqGGSbaBcqGGSbaBcqqGOaakcqWGObaAcqqGPaqkcqGH9aqpcqqG0baDcqqGYbGCcqqG1bqDcqqGLbqzaeaaaeaacqqGHbqycqqGUbGBcqqGKbazcqqGGaaicqqGVbWBcqGH9aqpcqqGbbqqcqqGYbGCcqqGNbWzcqqGnbqtcqqGTaqlcqqGmbatcqqGpbWtcqqGdbWqaeaacqaIWaamcqGG6aGoaeaacqqGVbWBcqqG0baDcqqGObaAcqqGLbqzcqqGYbGCcqqG3bWDcqqGPbqAcqqGZbWCcqqGLbqzaaaacaGL7baacqGGUaGlaaa@88CD@

Here, "current_node_ends_with_cell(*h*)" is a binary function that returns a true value if the current node in the history, *h*, ends with "cell". Given a set of features and a training corpus, the ME estimation process produces a model in which every feature *f*_*i *_has a weight *α*_*i*_. Like [25], we compute the conditional probability as follows:





The probability is calculated by multiplying the weights of the active features (i.e., those with *f*_*i *_(*h*,*o*) = 1); and *α*_*i *_is estimated by a procedure called Generalized Iterative Scaling (GIS) [37]. The ME estimation technique guarantees that, for every feature *f*_*i*_, the expected value of *α*_*i *_will equal the empirical expectation of *α*_*i *_in the training corpus. We use Zhang's MaxEnt toolkit and the L-BFGS [38] method of parameter estimation for our ME model.

### Baseline features

Table 15 shows the features used in our baseline argument classification model. Their effectiveness has been shown previously in [13,14,17,39]. Detailed descriptions of the features can be found in [19].

**Table 15:** The features used in the baseline argument classification model

**BASIC FEATURES**
• **Predicate **– The predicate lemma
• **Path **– The syntactic path through the parsing tree from the constituent being classified to the predicate
• **Constituent type**
• **Position **– Whether the phrase is located before or after the predicate
• **Voice **– passive if the predicate has a POS tag VBN, and its chunk is not a VP, or it is preceded by a form of "to be" or "to get" within its chunk; otherwise, it is active
• **Head word **– Calculated using the head word table described by Collins (1999)
• **Head POS **– The POS of the Head Word
• **Sub-categorization **– The phrase structure rule that expands the predicate's parent node in the parsing tree
• **First and last Word and their POS tags**
• **Level **– The level in the parsing tree
**PREDICATE FEATURES**
• **Predicate's verb class**
• **Predicate POS tag**
• **Predicate frequency**
• **Predicate's context POS**
• **Number of predicates**

**FULL PARSING FEATURES**
• **Parent, left sibling, and right sibling paths, constituent types, positions, head words, and head POS tags**
• **Head of Prepositional Phrase (PP) parent – **If the parent is a PP, then the head of this PP is also used as a feature

**COMBINATION FEATURES**
• **Predicate distance combination**
• **Predicate phrase type combination**
• **Head word and predicate combination**
• **Voice position combination**

**OTHERS**
• **Syntactic frame of predicate/NP**
• **Headword suffixes of lengths 2, 3, and 4**
• **Number of words in the phrase**
• **Context words & POS tags**

### Named entity features

In the English newswire domain, Surdeanu et al. [39] used NE features to indicate whether a constituent contains NEs of interest, such as personal names, organization names, location names, time expressions, and quantities of money. After adding these NE features to their system, the F-score improved by 2.12%. However, because NEs in the biomedical domain are quite different from English newswire NEs, we create specific biological NE features using the following five primary NE categories found in the GENIA ontology: protein, nucleotide, other organic compounds, source, and others. Table 16 lists the definitions of these five categories. When a constituent matches an NE exactly, the corresponding NE feature is enabled.

**Table 16:** Five NE categories in GENIA ontology

**NE**	**Definition**	**Abbreviation**
Protein	Proteins include protein groups, families, molecules, complexes, and substructures.	PTN
Nucleotide	A nucleic acid molecule or the compounds that consist of nucleic acids.	NUC
Other organic compounds	Organic compounds excluding proteins and nucleotides.	OOC
Source	Sources are biological locations where substances are found and their reactions take place.	SRC
Others	The terms that are not categorized as sources or substances can be marked.	OTR

### Biomedical template features

Although a few NEs tend to belong almost exclusively to certain argument types (e.g., "...cell" tends to be mainly an ArgM-LOC), this information alone is not sufficient for argument-type classification for two reasons: 1) most NEs appear in a variety of argument types; and 2), many appear in more than one constituent (a node in a parsing tree) in the same sentence. Take the sentence "IL4 and IL13 receptors activate STAT6, STAT3, and STAT5 proteins in the human B cells" for example. The NE "the human B cells" is found in two constituents ("the human B cells" and "in the human B cells") as shown in Figure 1. Yet only "in the human B cells" is annotated as ArgM-LOC because here "human B cells" is preceded by the preposition "in" and the determiner "the", which matches the template–"*IN *the *SRC*". Templates composed of NEs, words, and POS tags can be helpful for identifying the argument type of a constituent. In this section, we first describe our template generation algorithm, and then explain how we use the generated templates to improve SRL performance.

### Template generation (TG) and filtering

**Figure 6:**
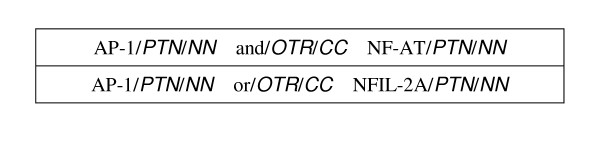
An aligned argument pair.

Our template generation (TG) algorithm, which extracts general patterns for all argument types using Smith and Waterman's local alignment algorithm [35], starts by pairing all arguments belonging to the same type according to their similarity. Closely matched pairs are then aligned word-by-word and a template satisfying the alignment result is created. Each slot in the template is given by the corresponding constraint information expressed in the form of a word (e.g. "the"), NE type, or POS. The preference of the constraint information is word > NE type > POS. If two aligned arguments have nothing in common for a given slot, the TG algorithm puts a wildcard in the position. Figure 6 shows a pair of aligned arguments, from which the TG algorithm generated the template "AP-1 *CC PTN*" (*PTN*: protein name) because, in the first position, both arguments have "AP-1"; in the second position, they have the same POS *CC*; and in the third position, they share a common NE type, *PTN*. The complete TG algorithm is described by pseudo code in Algorithm 1. The similarity function used to compare the similarity of two tokens in Smith and Waterman's algorithm is defined as:

Sim(x,y)=max⁡{1,x=y1,NE(x)=NE(y)0.8,POS(x)=POS(y)0,otherwise,
 MathType@MTEF@5@5@+=feaafiart1ev1aaatCvAUfKttLearuWrP9MDH5MBPbIqV92AaeXatLxBI9gBaebbnrfifHhDYfgasaacH8akY=wiFfYdH8Gipec8Eeeu0xXdbba9frFj0=OqFfea0dXdd9vqai=hGuQ8kuc9pgc9s8qqaq=dirpe0xb9q8qiLsFr0=vr0=vr0dc8meaabaqaciaacaGaaeqabaqabeGadaaakeaacqWGtbWucqWGPbqAcqWGTbqBcqGGOaakcqWG4baEcqGGSaalcqWG5bqEcqGGPaqkcqGH9aqpcyGGTbqBcqGGHbqycqGG4baEdaGabaabaeqabaGaeGymaeJaeiilaWIaemiEaGNaeyypa0JaemyEaKhabaGaeGymaeJaeiilaWIaemOta4KaemyrauKaeiikaGIaemiEaGNaeiykaKIaeyypa0JaemOta4KaemyrauKaeiikaGIaemyEaKNaeiykaKcabaGaeGimaaJaeiOla4IaeGioaGJaeiilaWIaemiuaaLaem4ta8Kaem4uamLaeiikaGIaemiEaGNaeiykaKIaeyypa0JaemiuaaLaem4ta8Kaem4uamLaeiikaGIaemyEaKNaeiykaKcabaGaeGimaaJaeiilaWIaem4Ba8MaemiDaqNaemiAaGMaemyzauMaemOCaiNaem4DaCNaemyAaKMaem4CamNaemyzaugaaiaawUhaaiabcYcaSaaa@7115@

where *x *and *y *are tokens in arguments *a*_*i *_and *a*_*j*_, respectively. The similarity of two arguments is calculated by the Smith and Waterman algorithm based on this token-level similarity function.

### Algorithm 1: Template generation

Input: A set of Arguments *A *= {*a*_1_,..., *a*_*k*_},

Output: A set of templates *T *= {*t*_1_,...,*t*_*k*_}.

1: *T *= {};

2: **for **each argument *a*_*i *_from *a*_1 _to *a*_*n*-1 _**do**

3:    **for **each argument *a*_*j *_from *a*_*i *_to *a*_*n *_**do**

4:       **if **the similarity of *a*_*i *_and *a*_*j *_calculated by an alignment is above the threshold *τ*

5:       **then **generate a common template ***t ***for *a*_*i *_and *a*_*j*_;

6:       *T*←*T*⋃***t***;

7:    **end**;

8: **end**;

After the templates have been generated, we filter out any template that matches at least two kinds of argument.

### Applying generated templates

The generated templates may match with constituents exactly or partially. In our experience, exact matches are more useful for argument classification. For example, constituents that perfectly match the template "*IN *a * *SRC*" ('*' means wildcards) are overwhelmingly ArgM-LOCs. Therefore, we only accept exact template matches. In other words, if a constituent matches a template *t *exactly, then the feature corresponding to *t *will be enabled.

## Appendix

1. **Agent**: deliberately performs the action (e.g., **Bill **drank his soup quietly).

2. **Patient**: experiences the action (e.g., The falling rocks crushed **the car**).

3. 

## Authors' contributions

RTH Tsai designed all the experiments and wrote most of this paper. WC Chou, ITH Yeh and YS Su discussed and refined the paper. YC Lin wrote the semantic role labelling programs and conducted all experiments. CL Sung wrote the template generation and matching programs. WC Chou, YS Su, and Wei Ku, the three biologists in our laboratory, annotated the BioProp corpus. TY Sung and WL Hsu guided the whole project.

**Figure 2:**
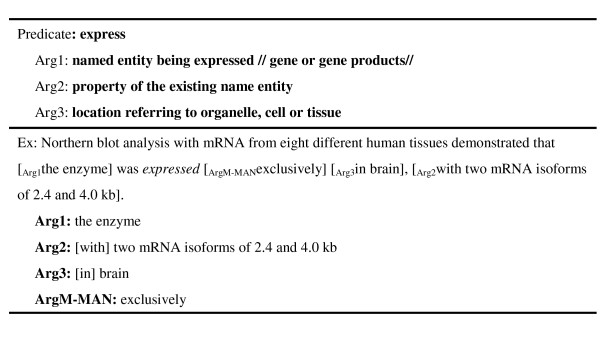
Frameset and annotated example of express defined in PASBio.
